# Effect of CD26/dipeptidyl peptidase IV on Jurkat sensitivity to G_2_/M arrest induced by topoisomerase II inhibitors

**DOI:** 10.1038/sj.bjc.6600791

**Published:** 2003-02-10

**Authors:** U Aytac, K Sato, T Yamochi, T Yamochi, K Ohnuma, G B Mills, C Morimoto, N H Dang

**Affiliations:** 1 Department of Lymphoma/Myeloma, MD Anderson Cancer Center, 1515 Holcombe Boulevard, Houston, TX 77030, USA; 2 Department of Clinical Immunology and AIDS Research Center, Institute of Medical Science-University of Tokyo, 4-6-1, Shirokanedai, Minato-ku, Tokyo 108-8639, Japan; 3 Department of Molecular Therapeutics, MD Anderson Cancer Center, 1515 Holcombe Boulevard, Houston, TX 77030, USA

**Keywords:** CD26/DPPIV, cell cycle, G_2_–M, topoisomerase II *α*, Jurkat

## Abstract

CD26/dipeptidyl peptidase IV (DPPIV) is a surface antigen with multiple functions, including a role in T-cell activation and the development of certain human cancers. We previously demonstrated that CD26/DPPIV enhanced sensitivity of Jurkat cells to doxorubicin. We now show that expression of CD26/DPPIV enhanced sensitivity of CD26 Jurkat transfectants to G_2_–M arrest mediated by the antineoplastic agent etoposide. The increased sensitivity to etoposide-induced G_2_–M arrest was associated with disruption of cell cycle-related events, including hyperphosphorylation of p34^cdc2^ kinase, change in cdc25C expression and phosphorylation, and alteration in cyclin B1 expression. CD26/DPPIV-associated enhancement of doxorubicin and etoposide-induced G_2_–M arrest was also observed in serum-free media, suggesting an effect of CD26 on cell-derived processes rather than serum-derived factors. Importantly, our work elucidated a potential mechanism for the enhanced susceptibility of CD26-expressing Jurkat cells to the topoisomerase II inhibitors by demonstrating that CD26/DPPIV surface expression was associated with increased topoisomerase II *α* levels and enhanced enzyme activity. Besides being the first to show a functional association between the multifaceted molecule CD26 and the key cellular protein topoisomerase II *α*, our studies provide additional evidence of a potential role for CD26 in the treatment of selected malignancies.

CD26 is a 110 kDa surface glycoprotein with diverse functional properties and is expressed on a variety of tissues, including selected lymphoid cells (Morimoto and Schlossman, 1998). Its extracellular domain encodes a membrane-associated dipeptidyl peptidase IV (DPPIV) activity, able to cleave amino-terminal dipeptides from polypeptides with either L-proline or L-alanine at the penultimate position, including chemokines involved in leukocyte function (Oravecz *et al*, 1997; Proost *et al*, 1998). CD26 is also involved in T-cell activation, owing to its association with CD45, mannose 6-phosphate/insulin-like growth factor II receptor and adenosine deaminase (ADA) (Morimoto *et al*, 1989; Dang *et al*, 1990a,1990b,1990c,1990d,^1991^; Torimoto *et al*, 1991; Kameoka *et al*, 1993; Morrison *et al*, 1993; Ikushima *et al*, 2000). Additionally, its DPPIV enzyme activity has a key role in various aspects of T-cell activation, as demonstrated by studies using DPPIV inhibitors, soluble CD26/DPPIV molecules or CD26 genetic mutants (Flentke *et al*, 1991; Tanaka *et al*, 1993,^1994^; Steinbrecher *et al*, 2001). Besides its involvement in normal T-cell function, CD26 may also have a role in the development of certain tumors (Tanaka *et al*, 1995; Stecca *et al*, 1997; Bauvois *et al*, 1999; Dang and Morimoto, 2002). For example, it is expressed on the surface of such aggressive T-cell malignancies as T-cell lymphoblastic lymphomas/acute lymphoblastic leukaemias and T cell CD30+ anaplastic large cell lymphomas, but not on the more indolent T-cell diseases like mycosis fungoides (Carbone *et al*, 1995; Jones *et al*, 2001).

Based on our hypothesis that CD26 expression influences tumour cell biology and potentially its sensitivity to cytotoxic treatments, as suggested by previous work showing that CD26 expression is associated with changes in tumour cell line behaviour *in vitro* and that the clinical behaviour of selected tumours may be correlated with differences in CD26 expression (Morrison *et al*, 1993; Carbone *et al*, 1995), we initiated studies using stable CD26 Jurkat transfectants to investigate the effect of CD26 on tumour sensitivity to selected antineoplastic agents. We recently demonstrated that Jurkat cells expressing CD26/DPPIV displayed enhanced sensitivity to cell cycle arrest induced by doxorubicin (Aytac *et al*, 2001). Owing to the potential implications of our findings with regards to future treatment of selected cancers, we investigated the effect of CD26/DPPIV on tumour sensitivity to etoposide, another key antineoplastic agent. In this paper, we extend our previous work by showing that expression of CD26/DPPIV resulted in enhanced sensitivity to etoposide-induced G_2_–M arrest, associated with such changes of key regulators of this checkpoint as hyperphosphorylation of p34^cdc2^, change in expression and phosphorylation of cdc25C and alteration in cyclin B1 expression. Furthermore, we demonstrate that the effect of CD26/DPPIV on drug sensitivity was independent of serum, as similar results were obtained in serum-free media. Finally and perhaps most importantly, we establish that expression of CD26/DPPIV resulted in enhanced levels of topoisomerase II *α* that correlated with increased enzyme activity, suggesting a potential mechanism for the observed increase in tumour sensitivity to the topoisomerase II inhibitors doxorubicin and etoposide.

## MATERIALS AND METHODS

### Cells and reagents

Human T-cell leukaemia Jurkat stable transfectants have been described (Tanaka *et al*, 1993; Aytac *et al*, 2001). The Jurkat cell lines include: (1) wild-type CD26-transfected Jurkat cell lines (wtCD26); (2) Jurkat cell lines transfected with mutant CD26 containing an alanine at the putative catalytic serine residue at position 630, resulting in a mutant CD26-positive/DPPIV-negative Jurkat transfectant (S630A); and (3) nontransfected parental Jurkat cells (parental). Jurkat transfectants were maintained in culture media, which consisted of RPMI 1640 supplemented with 10% FCS, penicillin (100 U ml^−1^), streptomycin (100 *μ*g ml^−1^), and G418 (0.25 mg ml^−1^) (Gibco BRL, NY, USA). Nontransfected parental Jurkat cells were maintained in the same culture media without G418. For certain experiments, AIM V serum-free media (Gibco, NY, USA) were used rather than culture media containing RPMI 1640 and 10% FCS. In experiments involving AIM V serum-free media, cells were washed extensively with sterile PBS, and then preincubated for 24 h at 37°C with AIM V-containing culture media to prevent contamination with serum. Following preincubation, cells were washed with sterile PBS, and then further incubate in AIM V-containing culture media with or without chemotherapeutic agents at the indicated doses. Anti-p34^cdc2^, anti-cdc25C and anti-cyclin B1 were from Santa Cruz (CA, USA), antitopo-isomerase II *α* (Ki-S1, recognising a carboxyterminal *α*-isozyme specific epitope) was from Boehringer Mannheim, antiactin was from Sigma, and the anti-CD26 antibody 1F7 was a murine antibody that has been described previously (Morimoto *et al*, 1989). Tetrazolium salt MTT (3,(4,5-dimethylthiazol-2-yl)2,5-diphenyltetrazolium bromide) (Sigma, MO, USA) was dissolved at a concentration of 5 mg ml^−1^ in sterile PBS at room temperature, with the solution being filter sterilized and stored at 4°C in the dark. Extraction buffer was prepared as follows: 20% w v^−1^ of SDS was dissolved at 37°C in a solution of 50% each of *N*,*N*-dimethyl formamide (DMF) (Sigma, MO, USA) and distilled water; pH was adjusted to 4.7 by the addition of 1 M HCl. Etoposide and doxorubicin were purchased from Calbiochem and was dissolved in sterile PBS. Hybond N^+^ membrane, [*α*-^32^P]dCTP and the Ready-To-Go DNA labelling beads were obtained from Amersham Biosciences. Human topoisomerase IIalpha gene probe YEpWob6 was kindly provided by Dr Leroy F Liu (Robert Wood Johnson Medical School, NJ, USA).

### MTT assay

Cell growth assay was performed as described previously (Aytac *et al*, 2001). Cells were incubated in microplates in the presence of culture media alone, culture media and doxorubicin or etoposide at the indicated concentrations for a total volume of 100 *μ*l (50 000 cells well^−1^). Following 36–48 h of incubation at 37°C, 25 *μ*l of MTT was added to the wells at a final concentration of 1 mg ml^−1^. The microplates were then incubated for 2 h at 37°C, followed by the addition of 100 *μ*l of extraction buffer. Following overnight incubation at 37°C, OD measurements at 570 nm were performed, with the s.e. of the mean of the triplicate wells being less than 15%. Cytotoxicity index was calculated as follows: 
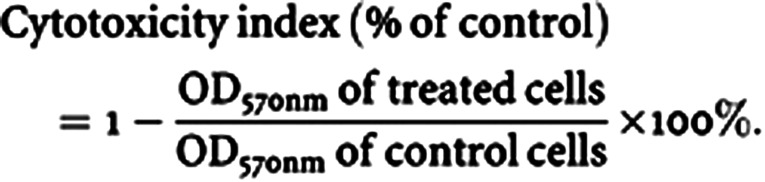


### Cell cycle analysis

Cells were incubated in culture media alone, culture media and doxorubicin or etoposide at indicated concentrations at 37°C for 24 h. Cells were then collected, washed twice with PBS and resuspended in PBS containing 10 *μ*g ml^−1^ propidium iodide, 0.5% Tween-20 and 0.1% RNase at room temperature for 30 min. Samples were then analysed (FACScan, Becton Dickinson, CA, USA) for DNA content. Cell debris and fixation artefacts were gated out and G_0_–G_1_, S, and G_2_–M populations were quantified using the CellQuest and ModFit LT programs.

### Sodium dodecyl – polyacrylamide gel electrophoresis (SDS-PAGE) and immunoblotting

After incubation at 37°C in culture media and doxorubicin or etoposide at the concentrations indicated for 24 h, cells were harvested from wells, washed with PBS and lysed in lysis buffer, consisting of 1% Brij 97, 5 mM EDTA, 0.02 M HEPES, pH 7.3, 0.15 M NaCl, 1 mM PMSF, 0.5 mM NaF, 10 *μ*g ml^−1^ aprotinin and 0.2 mM sodium orthovanadate. After incubating on ice for 15 min, nuclei were removed by centrifugation and supernatants were collected. 2×sample buffer consisting of 20% glycerol, 4.6% SDS, 0.125 M Tris, pH 6.8 and 0.1% bromophenol blue was added to the appropriate aliquots of supernatants. Following boiling, protein samples were submitted to SDS–PAGE analysis on an 8% gel under standard conditions using a mini-Protean II system (Bio-Rad, CA, USA). For immunoblotting, the proteins were transferred onto nitrocellulose (Immobilon-P, Millipore, MA, USA). Following overnight blocking at 4°C in a blocking solution consisting of 0.1% Tween-20 and 5% bovine serum albumin (BSA) in TBS, membranes were blotted with the appropriate primary antibodies diluted in blocking solution for 1 h at room temperature. Membranes were then washed with blocking solution, and appropriate secondary antibodies diluted in blocking solution were then applied for 1 h at room temperature. Secondary antibodies were goat anti-mouse or goat anti-rabbit HRP conjugates (Dako, CA, USA). Membranes were then washed with blocking solution and proteins were subsequently detected by chemiluminescence (Amersham Pharmacia Biotech, NJ, USA).

### Detection of DPPIV enzyme activity

Dipeptidyl peptidase IV (DPPIV) enzyme activity was detected by using an Enzyme Overlay Membrane system (EOM, Enzyme Systems Products, Dublin, CA, USA) to which the substrate Ala-Pro-AFC (7-amino-4-trifluoromethyl Coumarin) has been coupled, as described previously (Aytac *et al*, 2001). Following incubation at 37°C with media alone or media with etoposide for 24 h, cell lysates were prepared, and sample buffer consisting of 20% glycerol, 4.6% SDS, 0.125 M Tris, pH 6.8, 0.1% bromophenol blue and 2% 2-mercaptoethanol was added at room temperature. Samples were then submitted to SDS–PAGE analysis on an 8% gel under standard conditions. The EOM was moistened in 0.5 M Tris HCl, pH 7.8, placed over the surface of the gel, and incubated at 37°C for 40 min in a humidified atmosphere. The membrane was then removed from the gel and placed atop a long-wavelength UV lamp box to monitor enzymatic reaction, which involves the removal of the dipeptide Ala-Pro from the fluorogenic AFC and results in the appearance of fluorescent bands on the membrane.

### Immunofluorescence

All procedures were carried out at 4°C, and flow cytometry analyses were performed (FACScan, Becton Dickinson, CA, USA) as described previously (Dang *et al*, 1990d). Cells were stained with anti-CD26 antibody and washed two times with PBS and then with goat anti-mouse IgG FITC (Coulter). Cells were then washed two times with PBS prior to flow cytometry analyses. Negative controls were stained with second antibody alone.

### Preparation of nuclear extracts for detection of topoisomerase II *α*

For detection of topoisomerase II *α* by immunoblotting, isolation of nuclear fractions from Jurkat cells were prepared as follows. In brief, 10×10^6^ cells were harvested and allowed to swell for 15 min on ice in cytoplasmic extraction buffer (10 mM HEPES, 10 mM KCL, 0.1 mM EDTA, 0.1 mM EGTA, 1 mM DTT, 1 mM PMSF, 2 *μ*g ml^−1^ leupeptin, 2 *μ*g ml^−1^ aprotinin, and 0.5 mg ml^−1^ benzamidine). Then NP-40 (final concentration 0.3%) was added into that cell suspension and vortexed for 10 s. After 1 min of centrifugation at 16 000 **g**, the supernatant was removed. The pellet was then incubated with nuclear extraction buffer (20 mM HEPES, 400 mM KCL, 1 mM EDTA, 1 mM EGTA, 1 mM DTT, 0.5 mM PMSF, 2 *μ*g ml^−1^ leupeptin, 2 *μ*g ml^−1^ aprotinin, and 0.5 mg ml^−1^ benzamidine) for 30 min on ice with intermittent vortexing. The suspension was centrifuged at 16 000 **g** for 5 min, and supernatant was saved as the nuclear extract. SDS–PAGE and immunoblotting were then performed on the nuclear extracts. Each lane was equally loaded with 15 *μ*g of protein.

### Detection of topoisomerase II *α* activity

Topoisomerase II *α* catalytic activity was analysed through the use of the topoisomerase II decatenation assay kit according to the manufacturer's instructions (TopoGEN, Inc, OH, USA). Briefly, following incubation of Jurkat cells at 37°C for 24 h in culture media, 10×10^6^ cells were harvested and nuclear extracts were obtained. Appropriate dilutions of nuclear extracts of Jurkat CD26 transfectants or parental Jurkat cells were then incubated with 0.2 *μ*g of kinetoplast DNA (kDNA) in a 1×Topo II assay buffer (50 mM Tris-HCl, pH 8, 120 mM KCl, 10 mM MgCl_2_, 0.5 mM ATP, 0.5 mM dithiothreitol, and 30 *μ*g ml^−1^ BSA) for 60 min at 37°C. The reactions were terminated by the addition of 5×stop buffer/gel loading dye (final concentration: 1% Sarkosyl, 0.0005% bromophenol blue and 5% glycerol). DNA products were resolved on 1% agarose gel containing ethidium bromide (0.5 *μ*g ml^−1^) at 50 V for 60 min. After electrophoresis, the gel was destained in water for 30 min, and fluorescence was photographed by UV imager.

### Northern blotting studies

Total RNA was isolated from Jurkat cells by using Trizol Reagent (Life Technologies, NY, USA) according to the manufacturer's instructions. Then, RNA samples (20 *μ*g lane^−1^) were run on 1% agarose–formaldehyde gel and transferred to a Hybond N^+^ membrane for the Northern blot analysis by standard procedures. For detection of topoisomerase II *α* mRNA, YEpWob6 cDNA probe was labelled with [*α*-^32^P]dCTP using the Ready-To-Go DNA labelling beads (Amersham Biosciences, NJ, USA) and the blots were hybridised with the labelled probe in ULTRAhyb buffer (Ambion, TX, USA) for 24 h at 42°C. The blots were washed in 2×SSC and 0.1% SDS at 42°C and then washed in 0.1×SSC and 0.1% SDS at 42°C. The blots were exposed to a Phosphor Imager screen (Molecular Dynamics Storm MD 840, NJ, USA) for 24–48 h.

### Statistics

Student's *t*-test was used to determine whether the difference between control (S630A and parental Jurkat cells) and wtCD26 cells was significant (*P*<0.05 being significant).

## RESULTS

### Effect of CD26/DPPIV expression on etoposide-mediated growth inhibition and cell cycle arrest at the G_2_–M checkpoint of Jurkat cells

**Figure 1:**
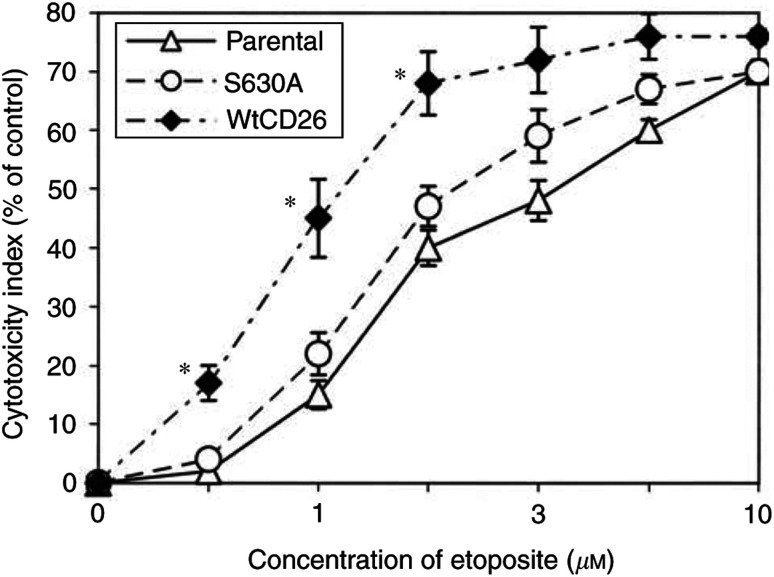
Effect of CD26/DPPIV expression on etoposide-mediated growth inhibition. CD26 Jurkat transfectants and parental cells were incubated at 37°C in culture media alone or culture media containing etoposide at the concentrations indicated, and MTT uptake assay was performed as described in Materials and Methods. wtCD26: wild-type CD26 Jurkat transfectant; S630A: CD26-positive/DPPIV-negative mutant CD26 Jurkat transfectant. Asterisks indicate wtCD26 samples with results significantly different from those of S630A and parental Jurkat cells (*P*<0.05). Data represent the means of three separate experiments. Cytotoxicity index was calculated as follows: 
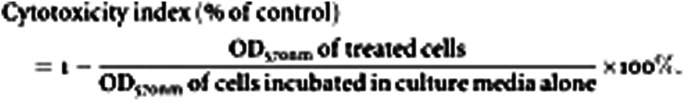

In view of our recent findings regarding the effect of CD26/DPPIV on sensitivity to the topoisomerase II inhibitor doxorubicin, we examined its effect on etoposide, another topoisomerase II inhibitor with antineoplastic activity, using stable CD26 transfectants of the Jurkat T-leukaemia cell line. Figure 1

shows that wtCD26 transfectants (wtCD26) displayed significantly increased sensitivity to etoposide compared to parental cells, as monitored by MTT uptake assays. Vector-only Jurkat transfectants also exhibited the same degree of drug sensitivity as parental cells (data not shown). Significantly, CD26 transfectants mutated at the DPPIV catalytic site (S630A) were also less sensitive to etoposide than wtCD26 transfectants. These data suggested that the presence of CD26, particularly its intrinsic DPPIV enzymatic activity, resulted in enhanced sensitivity to DNA damage mediated by the antineoplastic agent etoposide.

Cell cycle analysis by PI staining demonstrated that the enhancement in etoposide sensitivity seen with wtCD26 transfectants as compared to parental Jurkat cells was because of an increase in the percentage of cells arresting at G_2_–M (Table 1

**Table 1:** Enhanced etoposide-induced G2–M arrest associated with CD26/DPPIV

	**%G_0_–G_1_**	**%S**	**%G_2_–M**
*Media alone*			
Parental	49	38	13
S630A	47	42	11
wtCD26	46	40	14
*Etoposide (0.10 μM)*			
Parental	41	38	21
S630A	34	37	29
wtCD26	25	19	56
*Etoposide (0.25 μM)*			
Parental	27	32	41
S630A	22	30	48
wtCD26	12	19	69

CD26 Jurkat transfectants and parental cells were incubated at 37°C in media containing etoposide for 24 h. Cells were then harvested and cell cycle analyses were performed with PI staining. Data are representative of three separate experiments.

). Once again, DPPIV enzymatic activity was required to maximise drug sensitivity to etoposide as the G_2_–M block in the S630A CD26 mutant was significantly less than that seen with wtCD26 transfectants.

### CD26 expression and DPPIV enzyme activity on Jurkat transfectants

Data from Figure 2A

**Figure 2:**
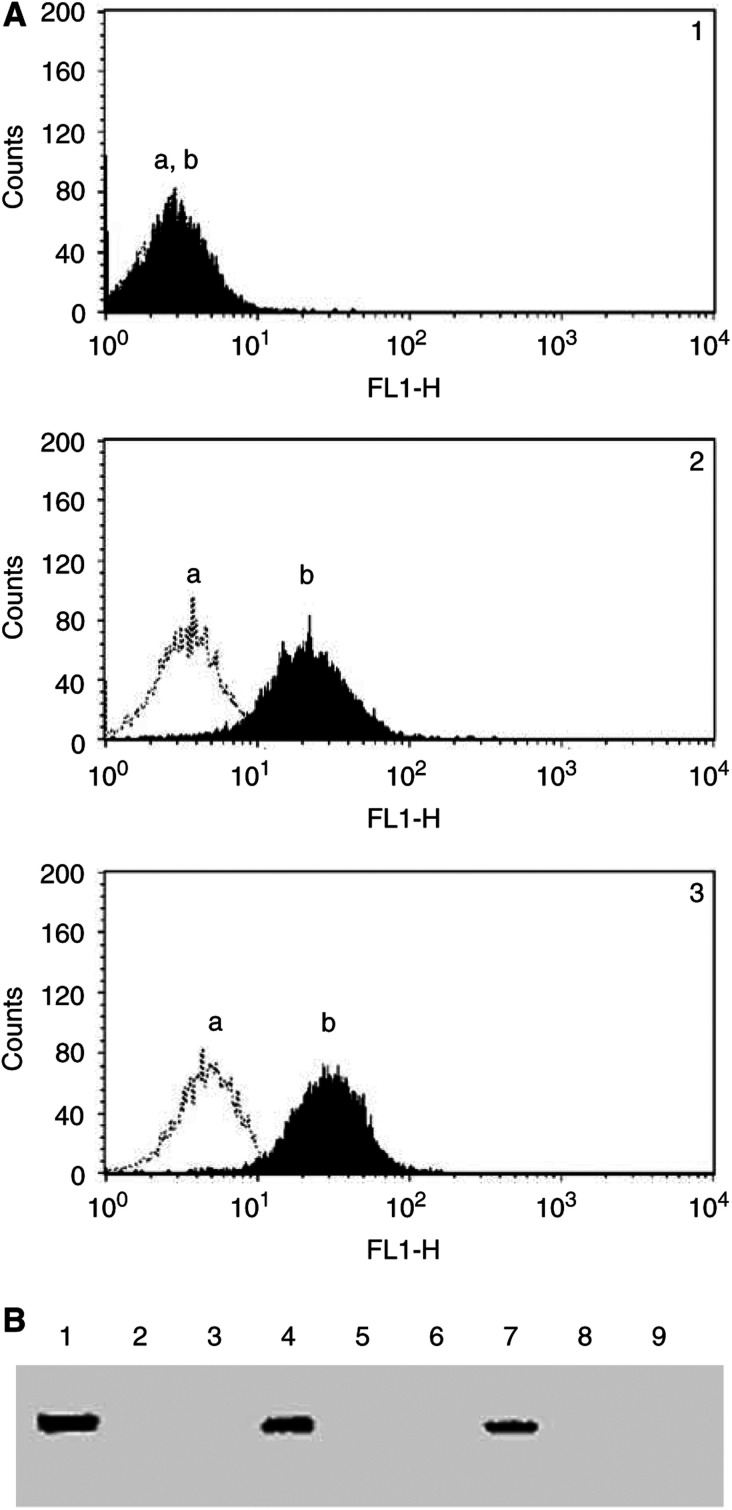
CD26 expression and DPPIV enzyme activity on Jurkat transfectants. (**A**) Jurkat cells were evaluated for CD26 expression by flow cytometry as described in Materials and Methods. panel 1: Parental Jurkat; panel 2: S630A transfectants; panel 3: wtCD26 transfectants; a: negative control; b: anti-CD26 antibody. (**B**) Jurkat cells were incubated for 24 h at 37°C with media alone or etoposide at 0.10 *μ*M or 0.50 *μ*M. Cells were then harvested, and DPPIV enzyme activity assays were performed as described in Materials and Methods. Lanes 1–3: media alone; lanes 4–6: etoposide at 0.10 *μ*M; lanes 7–9: etoposide at 0.50 *μ*M. Lanes 1, 4, 7: wtCD26; lanes 2, 5, 8: S630A; lanes 3, 6, 9: parental Jurkat cells.

showed that CD26 is expressed on the surface of wtCD26 and S630A Jurkat transfectants, and is not expressed on parental Jurkat cells. Figure 2B showed that only wtCD26 Jurkat transfectants expressed DPPIV enzyme activity, which still remains after treatment with etoposide. On the other hand, S630A transfectants as well as parental Jurkat cells did not exhibit DPPIV enzyme activity. These data therefore indicated that the observed differences in etoposide sensitivity in these various CD26 Jurkat transfectants were associated with differences in DPPIV enzymatic activity.

### Effect of CD26/DPPIV expression on p34^cdc2^, cyclin B1 and cdc25C following etoposide treatment of Jurkat cells

**Figure 3:**
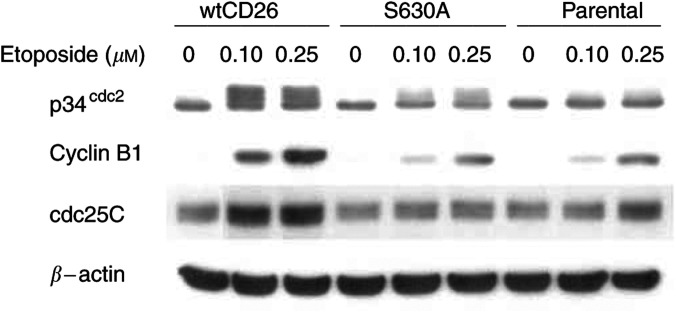
Effect of CD26/DPPIV expression on p34^cdc2^/cyclin B1 complex and cdc25C following etoposide treatment. Jurkat cells were incubated for 24 h at 37°C with media containing etoposide at the indicated doses. Cells were then harvested, and immunoblotting studies were performed with appropriate antibodies as described in Materials and Methods.

To elucidate the mechanisms involved in CD26/DPPIV-associated enhancement in etoposide-induced G_2_–M arrest, we evaluated the status of key regulators of this checkpoint (Figure 3

). p34^cdc2^ undergoes hyperphosphorylation at the inhibitory residues Thr14/Tyr15 following etoposide treatment, leading to decreased kinase activity associated with G_2_–M arrest (King *et al*, 1994; Poon *et al*, 1997; Aytac *et al*, 2001). As assessed by Western blot analysis, etoposide-treated wtCD26 Jurkat transfectants had higher levels of hyperphosphorylated p34^cdc2^ as compared to parental Jurkat cells and S630A transfectants, which only exhibited a slight enhancement in the level of phosphorylated p34^cdc2^ at the higher doses of etoposide.

Meanwhile, as demonstrated previously (Peng *et al*, 1997; Aytac *et al*, 2001), lysates from asynchronously growing Jurkat cells contained two major electrophoretic forms of cdc25C differing in serine-216 phosphorylation. Our studies consistently showed that wtCD26 transfectants displayed increased overall expression of cdc25C and enhancement in cdc25C serine-216 phosphorylation as compared to S630A transfectants and parental cells, when these cells were treated with etoposide. The increase in cdc25C serine-216 phosphorylation was therefore concordant with enhanced p34^cdc2^ inhibitory phosphorylation in etoposide-treated wtCD26 Jurkat transfectants as compared to parental cells or S630A transfectants.

Our studies also showed that the levels of cyclin B1, which associates with p34^cdc2^ as part of the key complex regulating cell cycle progression at the G_2_–M checkpoint, were higher in wtCD26 Jurkat transfectants as compared to those in parental cells or S630A transfectants following treatment with etoposide. As we previously noted (Aytac *et al*, 2001), we found in many experiments that cyclin B1 levels were slightly higher in untreated wtCD26 transfectants as compared to untreated parental cells or S630A transfectants. Nevertheless, treatment with etoposide consistently resulted in a significant rise in cyclin B1 level in wtCD26 transfectants, an increase greater than that seen in parental Jurkat cells or S630A transfectants. The relative level of cyclin B1 expression therefore correlated with the relative sensitivity of CD26 Jurkat transfectants to etoposide, similar to the case seen with p34^cdc2^ phosphorylation status and cdc25C serine-216 phosphorylation.

### Effect of CD26 expression on drug sensitivity was independent of serum

**Figure 4:**
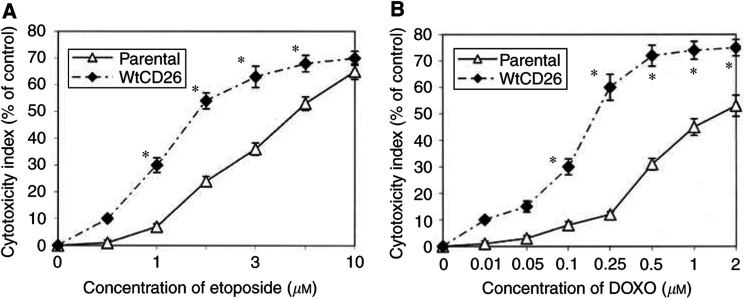
Enhanced sensitivity of wtCD26 to etoposide and doxorubicin in serum-free media. Following pretreatment with AIM V serum-free media at 37°C for 24 h, wtCD26 and parental Jurkat cells were incubated at 37°C in serum-free media containing etoposide (**A**) or doxorubicin (**B**) for 48 h, and MTT uptake assays were performed as described in Materials and Methods. Asterisks indicate wtCD26 samples with results significantly different from those of S630A and parental Jurkat cells (*P*<0.05). Data represent the means of three separate experiments. Cytotoxicity index was calculated as follows: 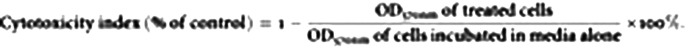

CD26 is able to cleave amino-terminal dipeptides from biological factors with either L-proline or L-alanine at the penultimate position through its DPPIV activity to alter their physiologic functions (Oravecz *et al*, 1997; Proost *et al*, 1998). To evaluate the possible role of serum-derived factors in CD26-associated enhancement in drug sensitivity, we incubated wtCD26 transfectants or parental Jurkat cells in AIM V serum-free media, and then performed similar studies as described above following doxorubicin or etoposide treatment. We demonstrated that the presence of CD26 still resulted in enhanced sensitivity to drug-induced G_2_–M arrest in serum-free media, as assessed by MTT uptake studies (Figure 4

) and cell cycle analyses (Table 2

**Table 2:** Enhanced sensitivity to etoposide and doxorubicin in serum-free media

	**%G_0_–G_1_**	**%S**	**%G_2_–M**
*Media alone*			
Parental	54	36	10
wtCD26	55	34	11
*Etoposide (0.25 μM)*			
Parental	51	30	19
wtCD26	34	23	43
*Etoposide (0.50 μM)*			
Parental	36	28	36
wtCD26	18	18	64
*Doxorubicin (0.10 μM)*			
Parental	43	30	27
WtCD26	31	20	49
*Doxorubicin (0.25 μM)*			
Parental	36	23	41
wtCD26	14	12	74

**Figure 5:**
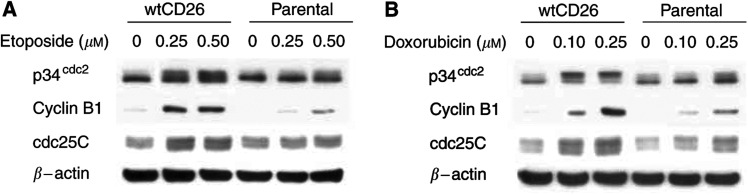
Effect of CD26 expression on G2/M checkpoint regulators following drug treatment in serum-free media. Following pretreatment with AIM V serum-free media at 37°C for 24 h, wtCD26 and parental Jurkat cells were incubated at 37°C in serum-free media containing etoposide (**A**) or doxorubicin (**B**) for 24 h at 37°C. Cells were then harvested and immunoblotting studies were conducted with appropriate antibodies as described in Materials and Methods.

Following pretreatment with AIM V serum-free media at 37°C for 24 h, wtCD26 Jurkat transfectants and parental cells were incubated at 37°C in serum-free media with etoposide or doxorubicin for 24 h. Cells were then harvested and cell cycle analyses were performed with PI staining. Data are representative of three separate experiments.

). Similarly, wtCD26 Jurkat transfectants exhibited greater p34^cdc2^ phosphorylation, overall expression and serine-216 phosphorylation of cdc25C, and cyclin B1 level when treated with etoposide or doxorubicin as compared to parental Jurkat cells (Figure 5

). Hence, these findings suggested that the enhanced drug sensitivity associated with CD26/DPPIV expression in CD26 Jurkat transfectants is because of its effect on cell-derived processes rather than because of its interaction with serum-derived factors.

### CD26/DPPIV expression is associated with enhanced topoisomerase II *α* level and activity

**Figure 6:**
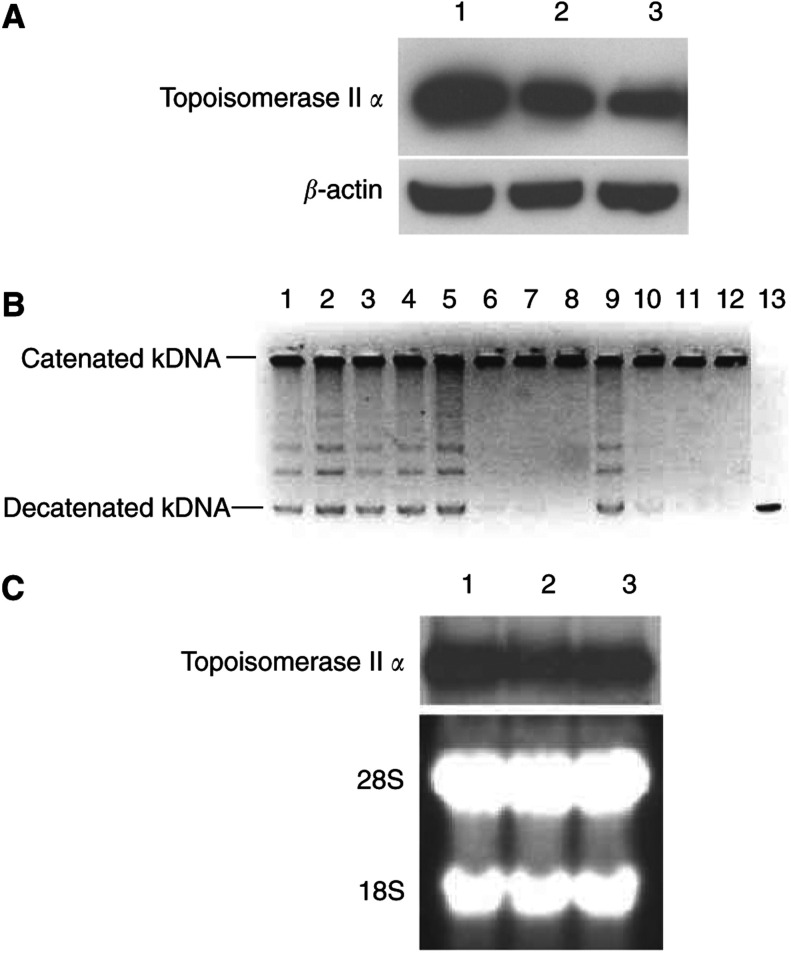
Topoisomerase II *α* expression, catalytic activity and mRNA level associated with CD26/DPPIV. (**A**) Jurkat cells were incubated in normal culture media, and nuclear extracts were collected for immunoblotting studies to evaluate topoisomerase II *α* protein levels as described in Materials and Methods. Each lane was equally loaded with 15 *μ*g of protein. Lane 1: wtCD26; lane 2: S630A cells; lane 3: parental Jurkat. Data are representative of three separate experiments. (**B**) Topoisomerase II decatenation assay was performed as described in Materials and Methods, with nuclear extracts from wtCD26 transfectants, S630A transfectants or parental Jurkat cells being assayed at the indicated concentrations. Lanes 1–4: wtCD26 Jurkat transfectants; lanes 5–8: S630A Jurkat transfectants; lanes 9–12: parental Jurkat cells. Lanes 1, 5, 9: 0.85 *μ*g of nuclear extracts; lanes 2, 6, 10: 0.75 *μ*g of nuclear extracts; lanes 3, 7, 11: 0.65 *μ*g of nuclear extracts; lanes 4, 8, 12: 0.50 *μ*g of nuclear extracts; lane 13: decatenated kDNA marker. Data are representative of three separate experiments. (**C**) Total cellular RNA (20 *μ*g) was isolated from Jurkat cells and subjected to Northern blot analysis with a ^32^P-radiolabelled topoisomerase II *α* cDNA probe as described in Materials and Methods. Lane 1: wtCD26 Jurkat; lane 2: S630A; lane 3: parental Jurkat. The lower panel represents ethidium bromide staining of RNA gel prior to transfer. Data are representative of three separate experiments.

As antineoplastic agents with key role in the treatment of haematological malignancies, doxorubicin and etoposide both target topoisomerase II *α* (Burden and Osheroff, 1998). To further explore the potential mechanism of CD26/DPPIV-associated enhancement in drug sensitivity, we evaluated topoisomerase II *α* expression in CD26 Jurkat transfectants. We found that wtCD26 Jurkat transfectants expressed higher level of topoisomerase II *α* than parental Jurkat cells or S630A transfectants (Figure 6A

), showing that the increased drug sensitivity in Jurkat cells expressing CD26/DPPIV was associated with enhanced topoisomerase II *α* level. Furthermore, we demonstrated that the enhanced topoisomerase II *α* protein level correlated with increased topoisomerase II *α* enzyme activity in wtCD26 Jurkat cells as compared to S630A transfectants or parental Jurkat cells. While decatenated DNA was observed with high concentrations of nuclear extracts from wtCD26, S630A and parental Jurkat cells, it was detected only in wtCD26 nuclear extracts at lower concentrations (Figure 6B). On the other hand, Northern blotting studies consistently detected similar levels of topoisomerase II *α* mRNA in wtCD26, S630A and parental Jurkat cells (Figure 6C).

## DISCUSSION

In this paper, we extended our earlier work by showing that the presence of DPPIV enzymatic activity of CD26-enhanced sensitivity of the human T-leukaemia line Jurkat to etoposide-induced DNA damage. Similar to our previous findings with the antineoplastic agent doxorubicin (Aytac *et al*, 2001), we demonstrated that etoposide exhibited greater effect on wtCD26 transfectants than parental Jurkat cells or CD26-positive/DPPIV-negative Jurkat transfectants through its effect on major regulators of the G_2_–M checkpoint, including p34^cdc2^, cdc25C and cyclin B1. We also showed that CD26-associated enhancement in sensitivity to doxorubicin and etoposide was independent of serum-derived factors, since similar effects were seen when cells were incubated in the presence of serum-free media. Significantly, our studies elucidated a potential mechanism involved in CD26/DPPIV-associated susceptibility to doxorubicin and etoposide-induced G2/M arrest by revealing a correlation between topoisomerase II *α* activity and protein level and CD26-associated sensitivity to the topoisomerase II inhibitors. However, we detected similar levels of topoisomerase II *α* mRNA in wtCD26, S630A and parental Jurkat cells. Results of our Northern blotting studies therefore suggested that the observed enhancement in topoisomerase II *α* level seen in Jurkat transfectants expressing CD26/DPPIV was not because of an overall increase in the steady-state level of topoisomerase II *α* mRNA. Topoisomeras II *α* levels are regulated through a number of mechanisms by various factors, including transcriptional rate, post-transcriptional alterations and post-translational modifications (Isaacs *et al*, 1998). Our laboratory is now actively conducting work to elucidate the particular mechanism(s) involved in CD26/DPPIV-associated enhancement in topoisomerase II *α* protein level and enzyme activity.

Essential for cellular proliferation as a key regulator of mitosis, DNA topoisomerase II enzyme catalyses many types of interconversions between different DNA topological isomers (Wang, 1996). Eukaryotes have two isoforms of topoisomerase II, *α* and *β*, which are coded by two distinct genes (Drake *et al*, 1989). A key factor in drug resistance is the intracellular level of topoisomerase II *α*, as several studies demonstrated that increased enzyme level is correlated with increased sensitivity, and drug resistance is often associated with decreased topoisomerase II *α* level (Davies *et al*, 1988; Fry *et al*, 1991; Larsen and Skladanowski, 1998). Consistent with these previous findings, our work showed that expression of CD26/DPPIV in Jurkat transfectants was associated with higher level of topoisomerase II *α*. While these findings suggested that a mechanism for the enhanced sensitivity to doxorubicin and etoposide was the increased intracellular level of their target enzyme, our data did not necessarily exclude the potential involvement of other additional factor(s) (Beck *et al*, 1993; Larsen and Skladanowski, 1998). Furthermore, a functional association between CD26 and topoisomerase II *α* may also prove to be an important and generalised aspect of CD26 biology. In fact, this interaction may play a role in CD26 function in T-cell physiology, particularly in view of well-established findings from multiple groups demonstrating CD26 involvement in T-cell activation and proliferation.

CD26/DPPIV cleaves selected cytokines at the NH_2_ terminus to alter their biological functions, resulting in lower chemotactic potency, impaired signalling effects and altered receptor specificity (Oravecz *et al*, 1997; Proost *et al*, 1998). Our studies with serum-free media showed that the presence of CD26/DPPIV was still associated with enhanced sensitivity to doxorubicin and etoposide, by influencing the key components of the G_2_–M checkpoint following drug treatment. These findings therefore suggested that CD26/DPPIV affects certain intrinsic cell-derived factors, independent of serum, to interfere with selected intracellular pathways, leading to enhanced drug sensitivity.

Previous studies have demonstrated that CD26 is expressed on the surface of selected aggressive T-cell malignancies (Carbone *et al*, 1995; Jones *et al*, 2001). Meanwhile, topoisomerase II *α* is highly expressed on aggressive B-cell non-Hodgkin's lymphomas; on the other hand, its expression on T-cell malignancies appears to be generally low, although this conclusion is based on studies with limited samples (Holden *et al*, 1995; Turley *et al*, 1997; Korkolopoulou *et al*, 2001). Our current data with the human T-cell leukaemia cell line Jurkat suggest that the expression of CD26 may be potentially linked to high topoisomerase II *α* level in selected T-cell malignancies, which are generally aggressive and often difficult to treat at the present time. These findings therefore suggest a correlation between CD26 and topoisomerase II *α* expression in haematological malignancies as well as perhaps selected solid tumours, which may also have potential clinical implications in view of the use of topoisomerase II inhibitors as major components of the treatment of these diseases. Along with our recent study demonstrating *in vitro* and *in vivo* antitumour effect of anti-CD26 antibody (Ho *et al*, 2001), our current work may therefore provide additional insights into the potential treatment strategies for selected cancers based on CD26 biology.
